# Adjuvanting a viral vectored vaccine against pre-erythrocytic malaria

**DOI:** 10.1038/s41598-017-07246-0

**Published:** 2017-08-04

**Authors:** Anita Milicic, Christine S. Rollier, Choon Kit Tang, Rhea Longley, Adrian V. S. Hill, Arturo Reyes-Sandoval

**Affiliations:** 1 The Jenner Institute, Nuffield Department of Medicine, University of Oxford, Old Road Campus Research Building, Roosevelt Drive, Oxford, OX3 7DQ UK; 2 Oxford Vaccine Group, Department of Paediatrics, University of Oxford and the NIHR Oxford Biomedical Research Centre, Centre for Clinical Vaccinology and Tropical Medicine, Churchill Hospital, Oxford, OX3 7LE UK; 3 The Jenner Institute, Nuffield Department of Medicine, University of Oxford, The Henry Wellcome Building for Molecular Physiology, Roosevelt Drive, Oxford, OX3 7BN UK; 4 Present Address: Duke-NUS Medical School, 8 College Road, Singapore, Singapore; 5 Present Address: Population Health and Immunity, Walter & Eliza Hall Institute, 1G Royal Parade, Parkville, VIC 3052 Australia and Mahidol Vivax Research Unit (MVRU) Faculty of Tropical Medicine, Mahidol University, Bangkok, 10400 Thailand

**Keywords:** Infectious diseases, Adjuvants

## Abstract

The majority of routinely given vaccines require two or three immunisations for full protective efficacy. Single dose vaccination has long been considered a key solution to improving the global immunisation coverage. Recent infectious disease outbreaks have further highlighted the need for vaccines that can achieve full efficacy after a single administration. Viral vectors are a potent immunisation platform, benefiting from intrinsic immuno-stimulatory features while retaining excellent safety profile through the use of non-replicating viruses. We investigated the scope for enhancing the protective efficacy of a single dose adenovirus-vectored malaria vaccine in a mouse model of malaria by co-administering it with vaccine adjuvants. Out of 11 adjuvants, only two, Abisco^®^-100 and CoVaccineHT^TM^, enhanced vaccine efficacy and sterile protection following malaria challenge. The CoVaccineHT^TM^ adjuvanted vaccine induced significantly higher proportion of antigen specific central memory CD8^+^ cells, and both adjuvants resulted in increased proportion of CD8^+^ T cells expressing the CD107a degranulation marker in the absence of IFNγ, TNFα and IL2 production. Our results show that the efficacy of vaccines designed to induce protective T cell responses can be positively modulated with chemical adjuvants and open the possibility of achieving full protection with a single dose immunisation.

## Introduction

Several new vaccines are urgently needed for diseases such as malaria, HIV-AIDS and tuberculosis, which, combined, kill about 4 million people every year, primarily in the developing countries^1^. Moreover, there is a need for potent vaccines that can achieve high protective efficacy with a single administration. This is in order to circumvent problems relating to the implementation of immunisation programmes, but also, importantly, to successfully contain and eradicate future pandemic outbreaks of infectious disease. As illustrated in the recent outbreak of the Ebola virus, an efficacious single dose vaccine would be a key tool in the early control and suppression of the spread of infection^2, 3^.

Replication-deficient human and chimpanzee adenoviruses (Ad) encoding a vaccine antigen are currently being actively evaluated in the development of prophylactic vaccines against a number of pathogens where T-cell responses are thought to play a protective role (*e*.*g*., malaria, HIV, hepatitis, influenza)^4–6^, and have more recently been applied in the development of a new Ebola vaccine^7^. Adenovirus-based vaccines were originally developed for their capacity to induce strong T-cell as well as B-cell responses in several animal models. The expectation is that the pathogen-associated molecular patterns naturally present in the recombinant adenovirus, and the resulting innate immune system activation, act as an intrinsic adjuvant and thus obviate the need for additional danger signals or chemical adjuvants as required for most sub-unit vaccines. However, despite the protection afforded in several animal models, efficacy has not always been satisfactory when translated to clinical trials^4^. This implies that, in parallel to vaccines based on sub-unit and recombinant proteins, there is still potential benefit in enhancing the immunogenicity and efficacy induced by viral vectors. One way of achieving this is by combining the vector with a vaccine adjuvant.

A well-characterized mouse model of malaria infection, based on *Plasmodium berghei*, is widely used for pre-clinical development and selection of novel vaccines against liver stage malaria^8, 9^. In particular, strong CD8^+^ cytotoxic responses are considered critical for protection against malaria challenge in this model^10, 11^, and these results have translated into humans^12^. The *P*. *berghei* challenge model therefore represents a good platform to investigate *in vivo* the immunological effects that different adjuvants may have on the immune response to the vaccine as well as on the protection afforded by a malaria-targeting adenovirus vaccine.

We selected a total of 11 different adjuvants and investigated their capacity to modify Ad vectored vaccine induced protection against malaria in the *P*. *berghei* challenge model. The vaccine was a chimpanzee Ad vector encoding the malaria antigen (Ag) insert ME.TRAP^5, 13^, which has been the most successful vectored malaria vaccine candidate to date and has shown good safety and efficacy both pre-clinically and in clinical trials^10, 12, 14^. The ME.TRAP insert contains a known *P*. *berghei* CD8^+^ T cell epitope, Pb9, which induces protective CD8^+^ T cell responses in mice^15, 16^ and is the basis for protection against malaria in this pre-clinical model. We investigated the adjuvant ability to enhance the virus vectored vaccine efficacy, and explored the immune responses underlying the increased protection in this murine model, revealing a fine-tuning of CD8^+^ T cell responses by different adjuvants.

## Results

### Abisco^®^-100 and CoVaccineHT^TM^ enhance the protective efficacy of a single dose Ad-ME.TRAP in a mouse model of malaria

**Figure 1:**
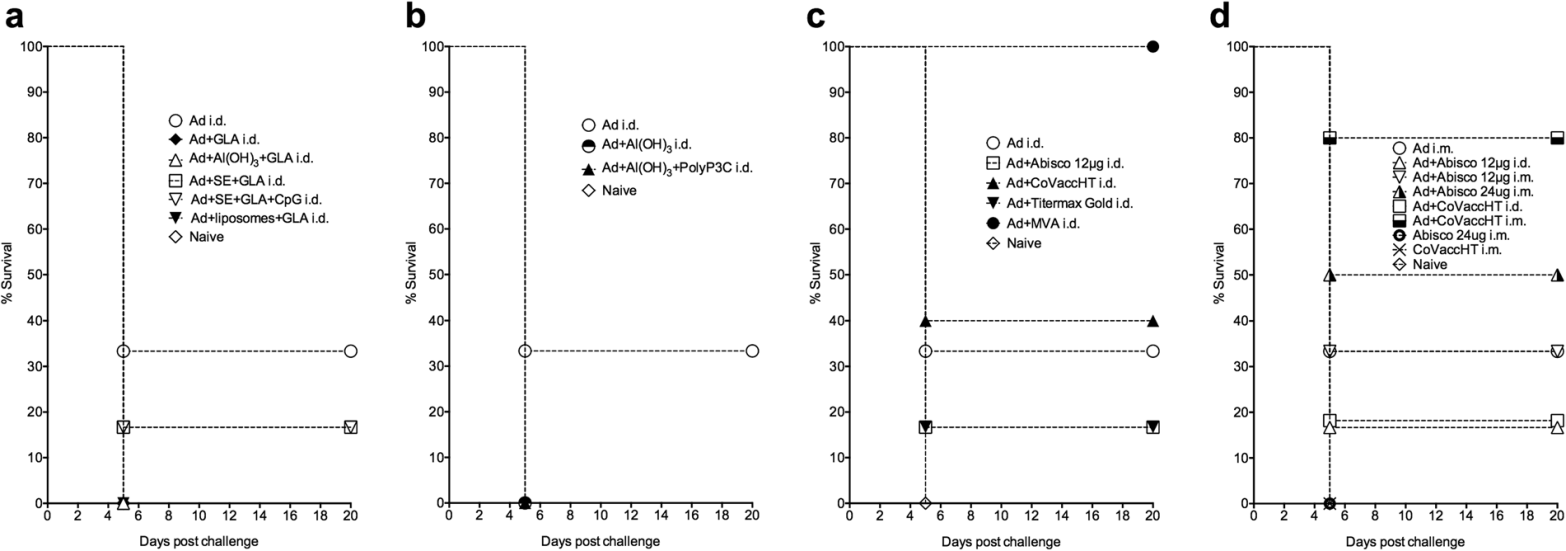
Survival graphs for mice challenged with malaria following immunisation with different Ad-ME.TRAP/adjuvant combinations. Balb/c mice (n = 6 to 10) were vaccinated with a single dose of Ad-ME.TRAP pre-erythrocytic malaria vaccine (5 × 10^9^ v.p./dose) alone or in combination with different adjuvants (**a** to **d**). Two weeks post-immunisation the animals were challenged i.v. with 1,000 *P*. *berghei* sporozoites and the vaccine efficacy measured by assessing parasitaemia in the blood following the challenge. Absence of parasitaemia at 3 weeks post-challenge indicated sterile protection from malaria. The baseline level of protection with the unadjuvanted Ad-ME.TRAP vaccine was around 30% (2/6 mice) in repeated experiments. Only two adjuvants, Abisco^®^-100 and CoVaccineHT^TM^ were able to enhance the vaccine efficacy. Each symbol is a median value from 6 animals (**a** to **c**), except in panel d where n = 10. Data in panels a–c were obtained over three experiments with several different adjuvants tested in each experiment. Panel d) shows results of one experiment; same results were obtained upon repeat.

The Ad vector used in this study was a live, non-replicating, chimpanzee adenovirus ChAd63 encoding ME.TRAP, from here on abbreviated to Ad-ME.TRAP. The vaccine was administered intradermally (i.d.) or intramuscularly (i.m.) to BALB/c mice at a sub-optimal dose of 5 × 10^9^ vp/animal, which was found to confer incomplete protection against malaria challenge. When given alone, the vaccine induced sterile protection in around a third of the vaccinated animals, allowing us to evaluate the effect of adding a chemical adjuvant (Fig. 1). We assessed a total of 11 different adjuvants or adjuvant combinations for their ability to enhance the vaccine protective efficacy: Alhydrogel (Al(OH)_3_), Glycolipid-A (GLA) – a TLR4 agonist, TiterMax^®^ Gold, Cholera Toxin (CT), Abisco^®^-100 (equivalent to Matrix M currently being developed by Novavax Inc., USA), CoVaccineHT^TM^ and combinations: Al(OH)_3_+GLA, Al(OH)_3_+Poly-Pam_3_Cys, Liposomes+GLA and stable emulsions of squalene-like oil in water: (SE)+GLA, SE+GLA+CpG. Further information on these adjuvants and their known mechanisms of action is provided in the Materials and Methods section. Co-administration of Ad-ME.TRAP with MVA-ME.TRAP served as a positive control for complete protection against malaria challenge^11^.

Two weeks after vaccination, the mice were challenged with a lethal dose of malaria by intravenous administration of 1,000 *P*. *berghei* sporozoites into the tail vein. We found that most of the tested adjuvants did not improve the vaccine protective efficacy, regardless of the immunisation route. Some, such as Al(OH)_3_, GLA, Poly-Pam_3_Cys and CT, apparently reduced the level of protection (Fig. 1a,b). In contrast, two multicomponent adjuvants, Abisco^®^-100 (at 24 μg/mouse) and CoVaccineHT^TM^ (given at 1 mg/mouse), were found to significantly increase sterile protection when co-administered with Ad-ME.TRAP via the i.m. route ﻿(Fig. 1c,d). CoVaccineHT^TM^ in particular was potent in inducing high protection, with 80% of the challenged animals sterilely protected. The adjuvants when administered alone had no protective efficacy against malaria (Fig. 1d).

**Figure 2:**
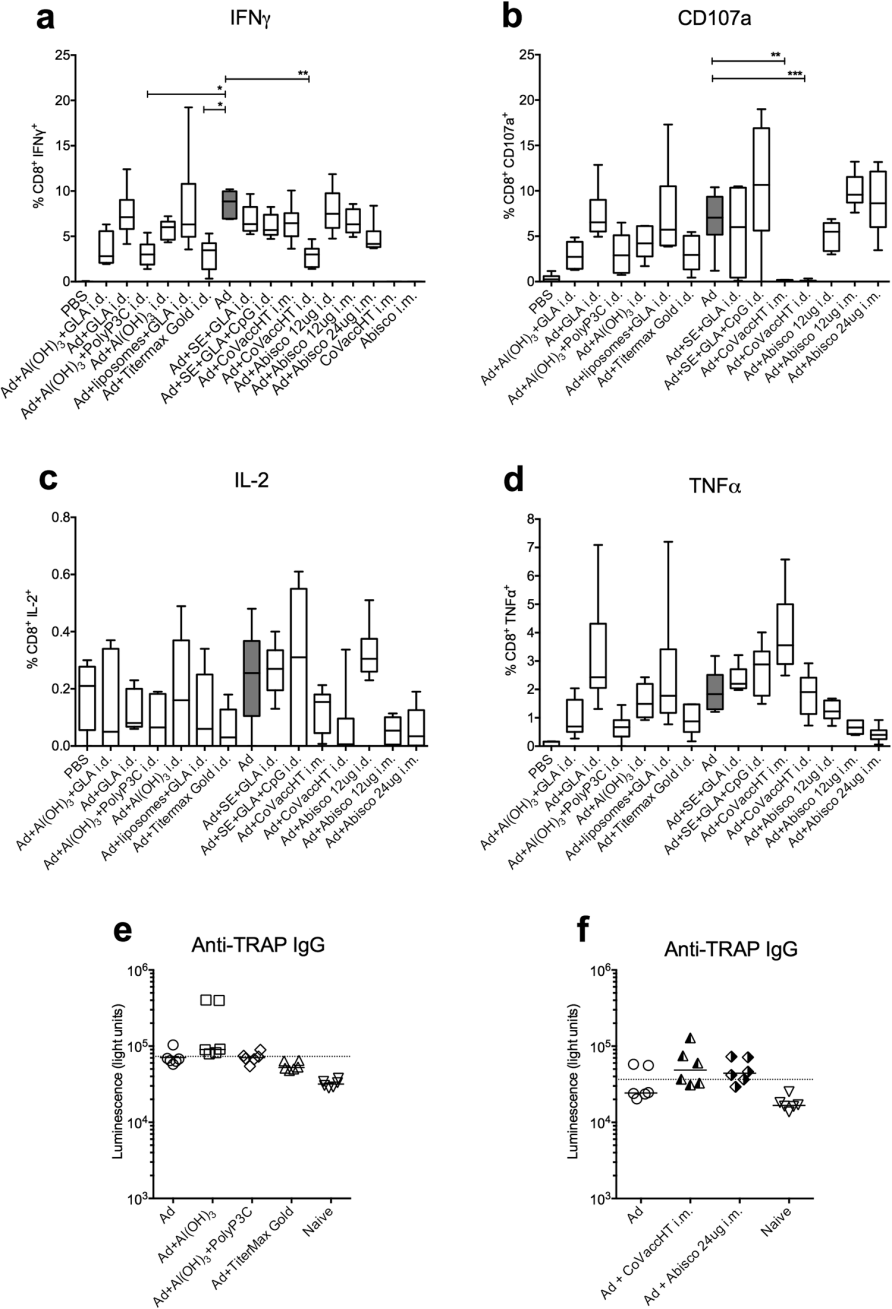
Antigen-specific cytokine producing CD8^+^ T cells and antibody titres in the peripheral blood of the immunised animals one day prior to malaria challenge. Two weeks after a single vaccination with different Ad-ME.TRAP/adjuvant combinations (Ad vector was given at 5 × 10^9^ v.p./dose), we assessed Pb9-specific intracellular cytokine levels in the peripheral blood of vaccinated Balb/c mice (n = 6 per group) (panels a–d). Isolated PBMCs were stimulated with Pb9 for 5 h and production of IFNγ, TNFα and IL2 and degranulation (CD107a expression) by CD8^+^ T cells assessed by ICS and flow cytometry. Ranked one-way ANOVA analysis (Kruskal-Wallis) with Dunn’s test for multiple comparisons was used to assess statistical significance of the differences between groups (*p < 0.05, **p < 0.01). Representative FACS dot plot data are shown in Supplementary Figure 2a. Panels e and f show total IgG antibody titres against the TRAP antigen in the serum of some of the vaccinated groups, measured two weeks post immunisation using the LIPS assay. The dashed line indicates the cut-off level of detection (average of 6 naïve samples plus 2 standard deviations).

We assessed the effect of the adjuvanted vaccine on Ag-specific CD8^+^ T cells and IgG antibody titres in peripheral blood of the vaccinated mice two weeks after the immunisation, i.e. one day before malaria challenge (Fig. 2): production of IFN-γ by CD8^+^ T cells following 5 h *in vitro* stimulation with the potent *P*. *berghei* mouse CD8^+^ T cell epitope Pb9, which is contained within the ME.TRAP insert, varied among the different Ad-ME.TRAP+adjuvant combinations, but was generally comparable to, or lower than, that induced by the Ad-ME.TRAP vaccine alone (Fig. 2a). A statistically significant reduction in the proportion of circulating Ag-specific CD8^+^IFN-γ^+^ cells was detected when Ad-ME.TRAP was given i.d. in combination with Al(OH)_3_+Poly Pam_3_Cys (p < 0.05), Al(OH)_3_+Titremax Gold (p < 0.05) and CoVaccineHT^TM^ (p < 0.01). Analysis of the cytotoxic T cell (CTL) degranulation marker CD107a in the PBMCs also showed variable levels among the animal groups vaccinated with Ad-ME.TRAP in different adjuvants, with the exception of CoVaccineHT^TM^ which appeared to completely abrogate the CD107a expression induced by the vaccine alone (Fig. 2b, p < 0.01 and p < 0.001 for i.m. and i.d. immunisations, respectively). The proportion of CD8^+^ cells producing IL-2 and TNFα did not significantly vary among the analysed groups (Fig. 2b and d). Total IgG antibody titres induced against the TRAP protein, as assessed by the Luciferase Immunoprecipitation System (LIPS) assay^17^, were low. However, measurable titres were found in the groups receiving Alhydrogel, Abisco^®^-100 and CoVaccineHT^TM^; no significant difference was found between the mice vaccinated with adjuvanted and unadjuvanted Ad-ME.TRAP (Fig. 2e,f).

### Early upregulation of IL-5 and IL-6 in the serum and single-positive CD8^+^CD107a^+^ T cells correlate with protection

**Figure 3:**
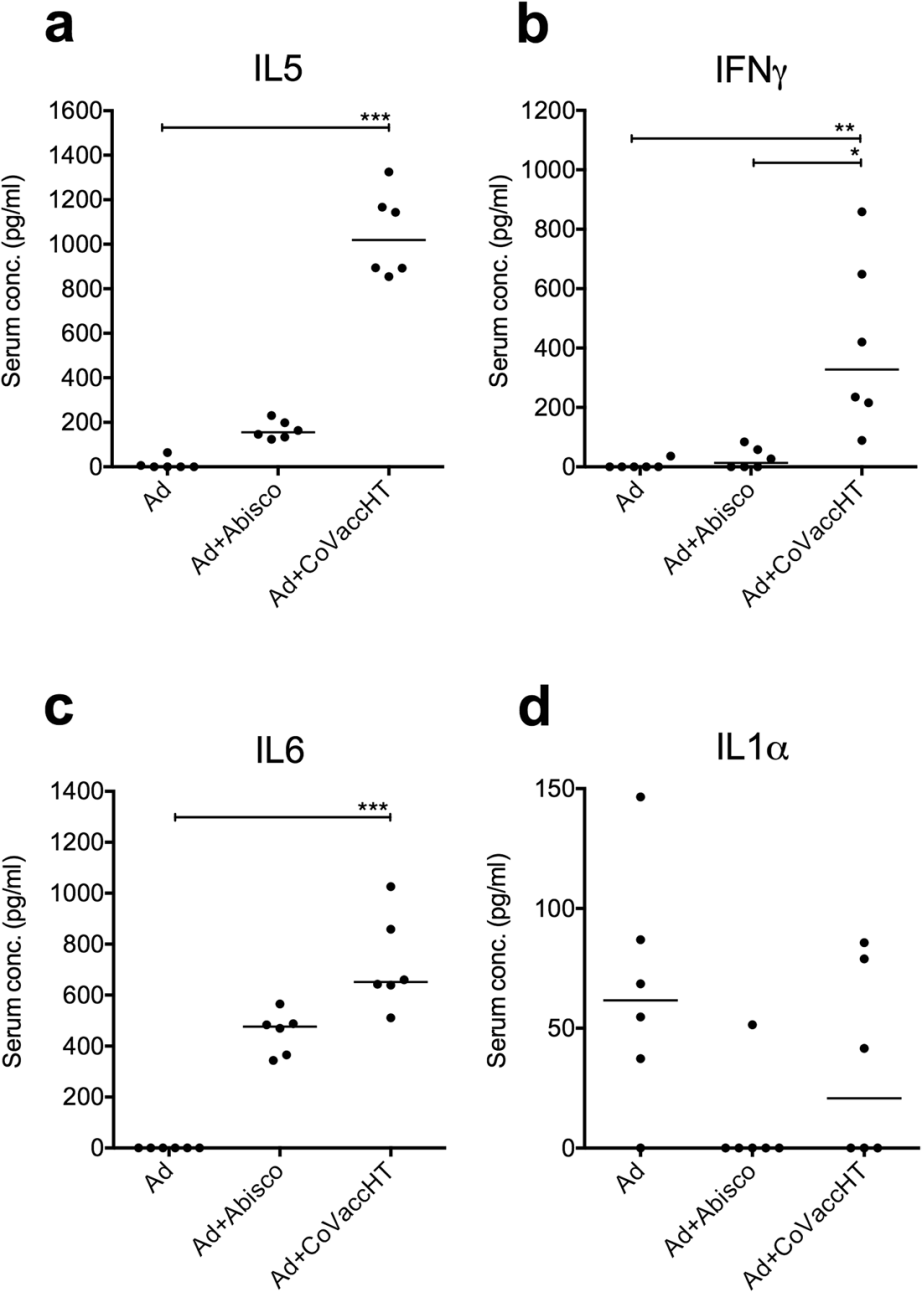
Analysis of serum cytokine levels in vaccinated animals. Serum samples from BALB/c mice (n = 6), vaccinated with Ad-ME.TRAP (5 × 10^9^ vp/dose) alone or adjuvanted with Abisco^®^-100 or CoVaccineHT, were collected at days 1, 7 and 14 post- immunisatiosn. Serum cytokine levels were measured using Th1/Th2/Th17/Th22 13-plex mouse multiplex bead array kit with added IL-1β. A number of cytokines could not be detected at any of the above time-points. Data are shown for cytokines that had differential levels of expression among the different experimental groups at day 1. Statistical analysis was carried out using ranked one-way ANOVA analysis (Kruskal-Wallis) with Dunn’s test for multiple comparisons; *p < 0.05, **p < 0.01, ***p < 0.001. Data from all three time-points for the detected cytokines are shown in the Supplementary Figure S1.

Cytokines circulating in the peripheral blood were assessed at 1, 7 and 14 days post i.m. vaccination with Ad-ME.TRAP adjuvanted with the two most protective adjuvants, Abisco^®^-100 and CoVaccineHT^TM^ (Supplementary Figure S1). Measurable levels of IFNγ, IL-1a, IL-1b, IL-2, IL-5, IL-6, IL-13, IL-27 were found, whereas IL-4, IL-10, IL-17, IL-21 and IL-22 were undetectable at any of the time-points studied. At 24 h post-vaccination (Fig. 3) a significantly higher level of IFN-γ was detected in the group receiving Ad-ME.TRAP+CoVaccineHT^TM^ (p < 0.001). Cytokines IL-5 and IL-6 were significantly up-regulated in the mice receiving the CoVaccineHT^TM^ -adjuvanted vaccine (p < 0.001) and were undetectable in the group receiving Ad-ME.TRAP alone (Fig. 3).

**Figure 4:**
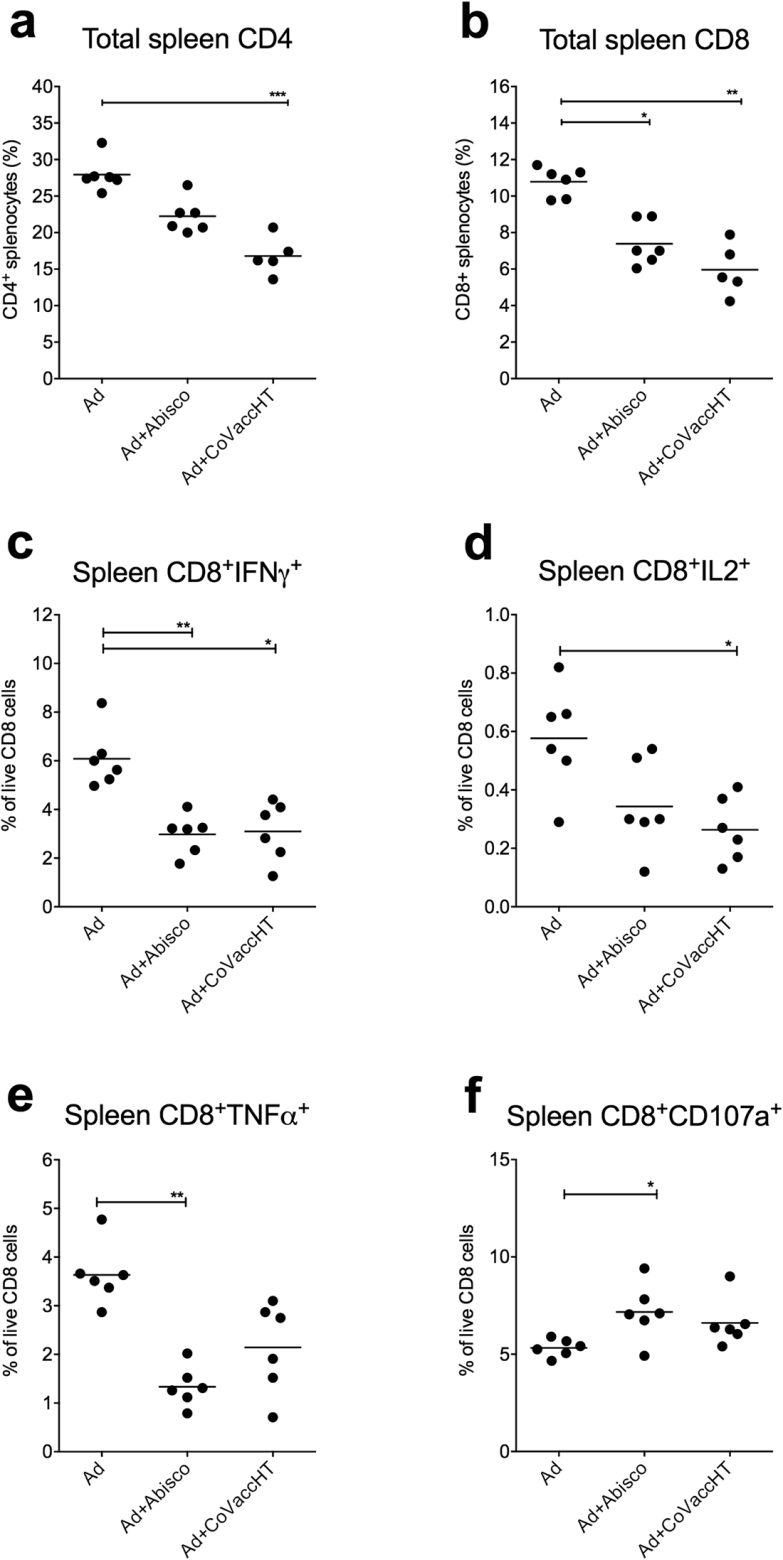
Splenocyte analysis in animals vaccinated with the adjuvanted and unadjuvanted Ad-ME.TRAP vaccine. Two weeks after immunisation of BALB/c mice (n = 6) with Ad-ME.TRAP (5 × 10^9^ vp/dose), alone or adjuvanted with Abisco^®^-100 or CoVaccineHT, relative proportion of different cell subsets in the spleen were analysed by flow cytometry. Data are shown for CD4 and CD8 T cells (panels a and b). In parallel, production of IFNγ, TNFα and IL2 and degranulation (CD107a expression) by CD8^+^ splenocytes, following 4 h of *in vitro* stimulation with Pb9 peptide, was assessed by intracellular staining and flow cytometry (**c–f**). Representative FACS dot plot data are shown in the Supplementary Figure S2b. Ranked one-way ANOVA analysis (Kruskal-Wallis) with Dunn’s test for multiple comparisons was used to assess statistical significance between groups; *p < 0.05, **p < 0.01, ***p < 0.001. Data shown are representative of three independent experiments.

Protective efficacy of Ad-ME.TRAP has previously been associated with CD8^+^ T cells^10, 12^. To investigate potential correlates of protection of the vaccine in combination with Abisco^®^-100 and CoVaccineHT^TM^, we first assessed the total number of different T cell populations (CD4^+^, CD8^+^, γδ) and NK cells in the spleen and peripheral blood at two weeks post i.m. immunisation. In the spleen, the proportion of CD4^+^ and CD8^+^ T cells was markedly decreased in the animals vaccinated with the adjuvanted vaccine, reaching statistical significance for both CD4^+^ (p < 0.001) and CD8^+^ (p < 0.01) T cells in the mice immunised with Ad-ME.TRAP+CoVaccineHT^TM^ (Fig. 4a,b). Comparable levels of γδ T cells were observed in all groups, while the number of NK cells was non-significantly elevated in the Ad-ME.TRAP+CoVaccineHT^TM^ group. Cytokine release by splenic CD8^+^ T cells after *in vitro* stimulation with Pb9 peptide paralleled the reduced CD8^+^ T cell numbers: mice that received the adjuvanted vaccine had a significantly lower proportion of CD8^+^IFNg^+^(p < 0.05 for Abisco^®^-100 and p < 0.01 for CoVaccineHT^TM^, Fig. 4c), CD8^+^IL2^+^ (p < 0.05 for CoVaccineHT^TM^, Fig. 4d) and CD8^+^TNFa^+^ (p < 0.01 for Abisco^®^-100, Fig. 4e), while CD8^+^ T cells expressing the degranulation marker CD107a showed a modest elevation with both adjuvants (p < 0.05 for Abisco^®^-100, Fig. 4f).

**Figure 5:**
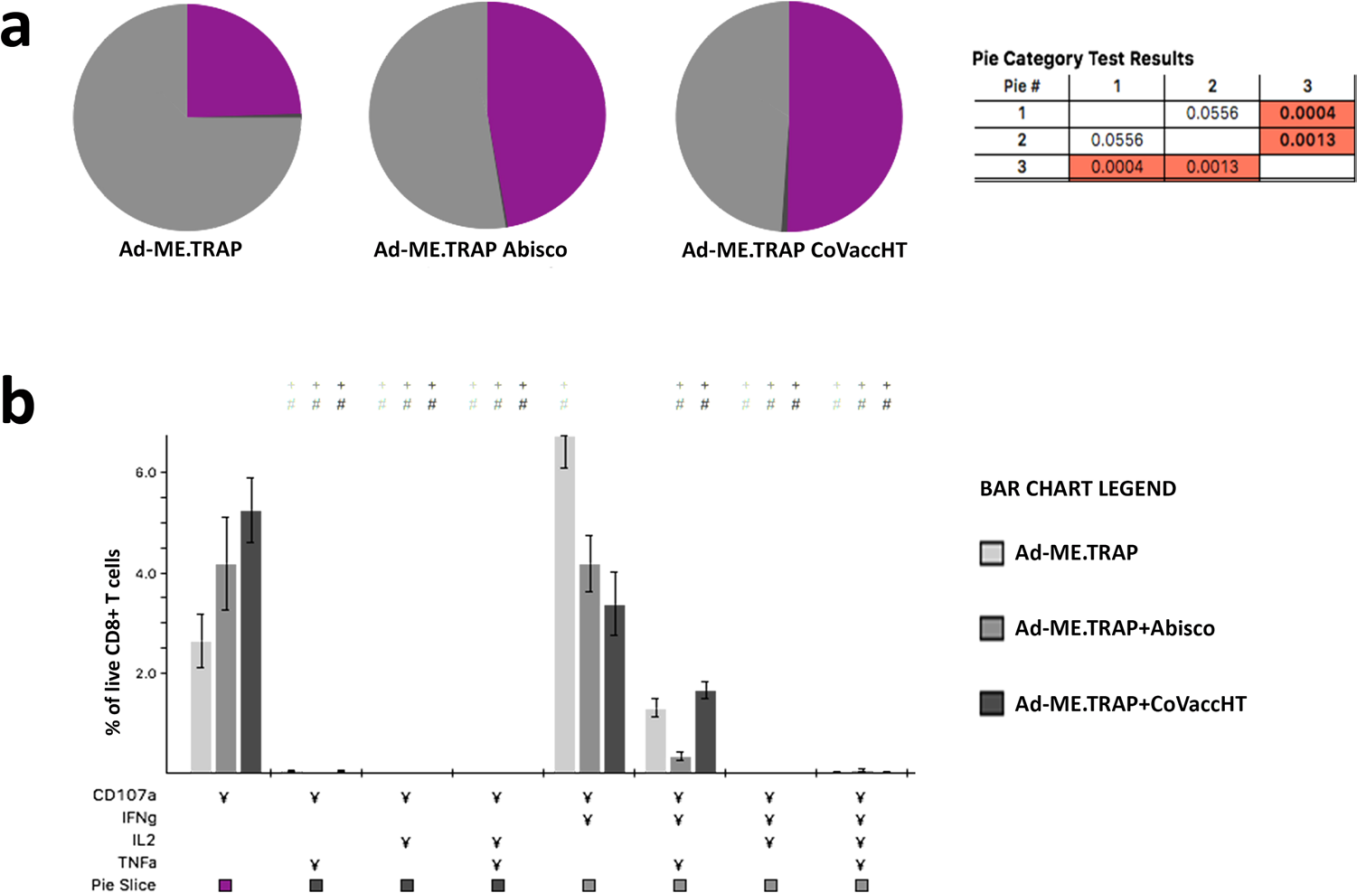
Functionality of CD8^+^ T cells: protective adjuvants induce higher levels of circulating CD8^+^CD107a^+^IFNγ^−^TNFα^−^IL2^−^ T cells. BALB/c mice (n = 6) were immunized intramuscularly with Ad-ME.TRAP (5 × 10^9^ vp/dose), alone or adjuvanted with Abisco^®^-100 or CoVaccineHT and the cytokine profiles of CD8^+^ PBMCs two weeks post-immunization analysed using the SPICE software^18^. (**a**) Pie charts show the proportion of PBMC CD8^+^ T cells producing one or more of: CD107a, IFNγ, TNFα and IL2 upon *in vitro* stimulation with the Pb9 peptide. Vaccine received is indicated under each pie-chart. Purple coloured area represents cells that are positive only for CD107a and negative for IFNγ, TNFα and IL2. A statistically significant distribution was found between the group receiving Ad-ME.TRAP+CoVaccineHT^TM^ and the other two groups (the p-values are shown in the Table next to the charts). (**b**) More detailed representation of the cytokine secreting profile among the vaccinated groups of mice. Each set of bars shows the proportion of CD8^+^ T cells secreting one or more cytokines (marked with ¥) in the three vaccinated groups.

A further investigation of the functionality of CD8^+^ T cells was performed using a Boolean analysis in FlowJo (FlowJo LLC, Oregon, USA) and data analysis in SPICE software^18^ to determine if any of the sub-populations of PBMC CD8^+^ T cells producing single cytokines or combinations of IFNγ, TNFα, IL-2 or CD107a after﻿ *in vitro* stimulation with Pb9 peptide﻿﻿ were upregulated upon vaccination. This analysis revealed an increase in the proportion of CD107a single positive cells in the peripheral blood following the administration of either adjuvanted vaccine (Fig. 5), while double or triple positive cells expressing IFNγ in addition to CD107a, showed a decrease in the presence of the adjuvant, supporting the observations shown in Fig. 2.

### Antigen-specific T_E_ and T_CM_ cells are up-regulated and T_EM_ cells down-regulated systemically in animals receiving the adjuvanted vaccine

**Figure 6:**
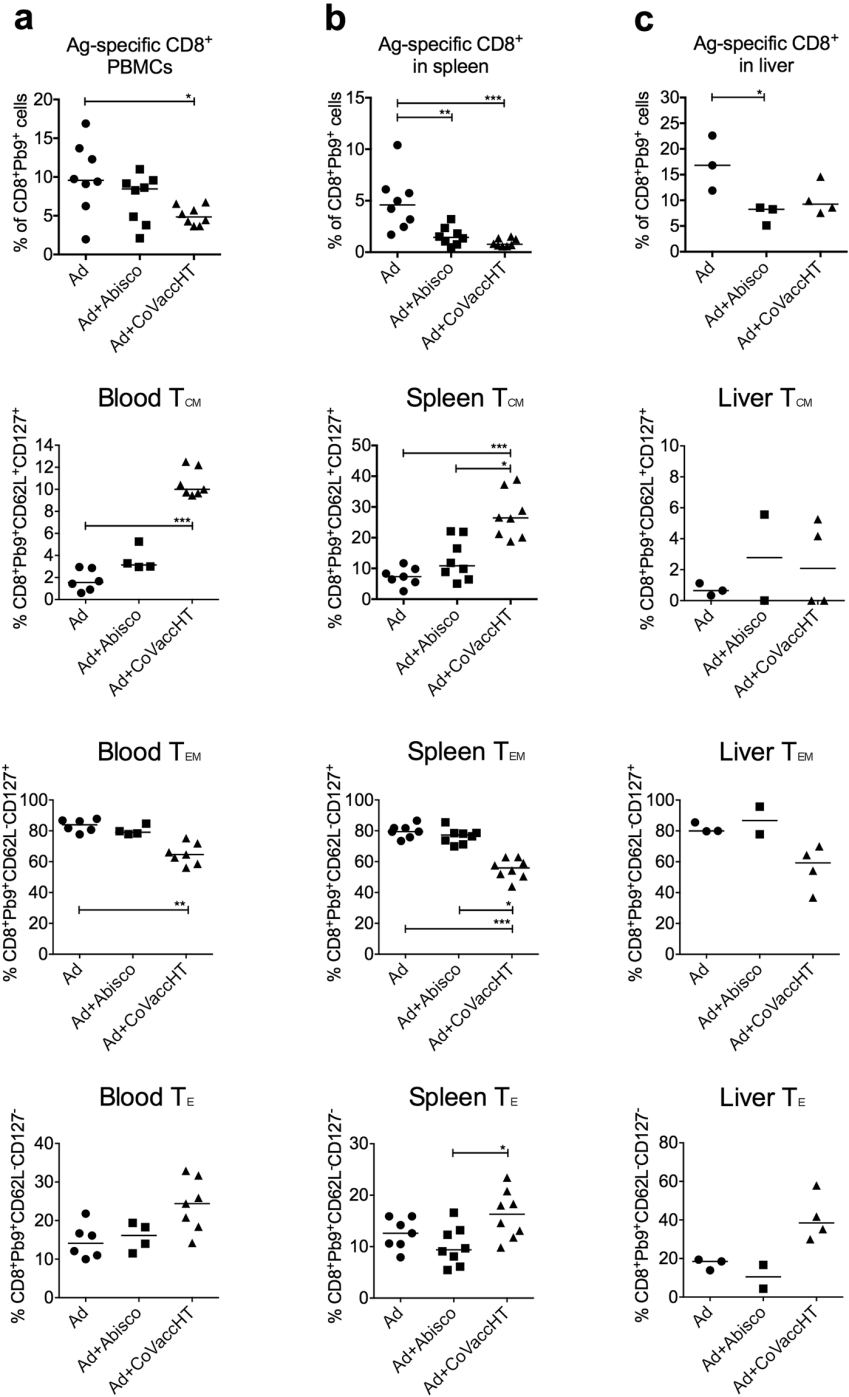
Memory profiles of CD8^+^ T cells in the peripheral blood, spleen and liver of animals vaccinated with unadjuvanted and adjuvanted Ad-ME.TRAP. BALB/c mice (n = 8) were immunized intramuscularly with Ad-ME.TRAP (5 × 10^9^ vp/dose), alone or adjuvanted with Abisco^®^-100 or CoVaccineHT. Proportion of Pb9-specific CD8^+^ T cells in the peripheral blood, spleen and liver, and their memory profiles (T_E_, T_EM_ and T_CM_) were evaluated by flow cytometry using Pb9-tetramer and surface markers CD127 and CD62L. The three memory cell subsets were defined as: T_E_ = CD62L^−^CD127^−^, T_EM_ = CD62L^−^CD127^+^ and T_CM_ = CD62L^+^CD127^+^. Representative FACS data are shown in the Supplementary Figure S2c. Ranked one-way ANOVA analysis (Kruskal-Wallis) with Dunn’s test for multiple comparisons was used to assess statistical significance of the differences between groups; *p < 0.05, **p < 0.01, ***p < 0.001.

We investigated the proportion and phenotype of Pb9-specific CD8^+^ T cells in the peripheral blood, spleen and liver of mice immunised i.m. with the protective adjuvant/vaccine combinations two weeks post-immunisation (corresponding to one day before challenge). A Pb9 tetramer and CD62L and CD127 cell surface markers were used to identify effector memory, central memory and effector T cells (T_EM_, T_CM_ and T_E_, respectively, Fig. 6), as reported previously^10^. In support of the results shown in Fig. 4, the proportion of Ag-specific CD8^+^ T cells was found to be lower at all three sites in mice vaccinated with the adjuvanted vaccine compared to the unadjuvanted Ad-ME.TRAP, with a statistically significant reduction detected with Ad-ME.TRAP+CoVaccineHT^TM^ in peripheral blood (p < 0.05) and spleen (p < 0.001) and with Ad-ME.TRAP+Abisco^®^-100 in spleen (p < 0.01) and liver (p < 0.05). Phenotypically, adjuvanting the vaccine had a pronounced effect on the Ag-specific CD8^+^ T cell memory subsets. Immunisation with Ad-ME.TRAP + CoVaccineHT^TM^ resulted in a significantly higher frequency of Ag-specific CD8^+^ T_CM_ cells in peripheral blood and the spleen than Ad-ME.TRAP alone (p < 0.001). The CD8^+^ T_E_ cell population in mice immunised with Ad-ME.TRAP+CoVaccineHT^TM^ was also increased in the three sites analysed, reaching significance in the spleen (p < 0.05, Fig. 6c). In contrast, the T_EM_ cell population was lower in this group compared to Ad-ME.TRAP alone and Ad-ME.TRAP+Abisco^®^-100 at all three analysed sites, reaching statistical significance in blood and spleen compared to Ad-ME.TRAP (P < 0.01 and p < 0.001, respectively, Fig. 6b). In the mice vaccinated with Ad-ME.TRAP+Abisco^®^-100, the T_CM_, T_E_ and T_EM_ populations in blood and spleen showed the same trend as with Ad-ME.TRAP+CoVaccineHT^TM^, but their frequencies were not significantly different from those in mice that received Ad-ME.TRAP alone.

## Discussion

Development of novel and highly protective subunit vaccines relies on the use of adjuvants to improve immunity against infectious diseases. We assessed 11 adjuvants and adjuvant combinations, in different stages of pre-clinical and clinical development, together with an adenoviral-vectored vaccine targeting the malaria pre-erythrocytic stage Ad-ME.TRAP, for an improved protective efficacy against sporozoite challenge. Classically used aluminium-based adjuvants induce good antibody responses but have limited capacity to stimulate cellular adaptive immunity^19^. As cellular immunity is a major component required to protect against intracellular pathogens, such as malaria, we focused on the effect of adjuvants on CD8^+^ T-cell mediated immunity in a murine model of malaria using an immunodominant MHC-I epitope from the *Plasmodium berghei* circumsporozoite protein, Pb9^20^.

A safe and efficacious vaccine that can provide full and lasting protection with a single dose is the ultimate goal in the development of all vaccines. Replication-deficient adenoviruses are known to be safe, potent and immunogenic vaccine delivery vectors. We sought to investigate whether higher protective efficacy of a single immunisation with an Ad-based vaccine, while retaining safety, can be achieved by the addition of an adjuvant.

We chose a dose of the Ad-ME.TRAP vaccine that provides partial protection against malaria in this model  (30% of the challenged animals were fully protected), so that any added benefit of the adjuvants could be observed. Due to the inherent immunogenicity of the virus, replication-deficient Ad-vectored vaccines are potent inducers of both the innate and the adaptive immune responses against the encoded Ag. Thus, unsurprisingly, most of the tested adjuvants did not enhance the protective efficacy of the Ad-ME.TRAP vaccine alone; some, such as cholera toxin and TLR4 agonist GLA even resulted in an apparent reduction in protection in this model. This observation further supports the recognised difficulties in adjuvanting vaccine delivery vehicles with intrinsic adjuvant properties, such as viral vectors. In our study, only two adjuvants, Abisco^®^-100 and CoVaccineHT^TM^, out of the 11 tested, enhanced the vaccine protective efficacy. Although of different composition, both of these adjuvants are complex compounds containing immunogenic components based on lipid, saponin or polysaccharide molecules, organised into cage-like structures (Abisco^®^-100) or oil-droplets (CoVaccineHT^TM^).

We assessed in detail the cellular immune responses accompanying the enhanced protection. Although these two very different adjuvants are likely to have different mechanisms of enhancing immunogenicity, we explored various immune readouts post-vaccination, looking for a potential common parameter induced by both.

A cytokine profile analysis indicated that the production of IL-5 and IL-6 increased within 24 h of vaccination with either adjuvant, with CoVaccineHT^TM^ showing a statistically significant difference. These two cytokines are known to stimulate antibody secretion by activated B cells^21^. We observed a half-log increase in Ag-specific antibody responses with the addition of CoVaccineHT^TM^ to Ad-ME.TRAP, which might provide some antibody-mediated protection in addition to the Pb9-specific CD8^+^ response. Elevated serum levels of IFNγ at 24 h in the group receiving CoVaccineHT^TM^ i.m. indicates early involvement of a Th1 response following this vaccination regimen. Production of both IL-5 and IFNγ following intramuscular injection of the vectored vaccine with CoVaccineHT^TM^ supports the idea of a balanced Th1/Th2 response, which could be due to a mixture of Th cells at different stages of the activation pathway, determined by the strength and duration of the signal delivered by antigen presenting cells^22^, and resulting from a complex interaction between the viral delivery vector and the CoVaccineHT^TM^ adjuvant. In addition we detected upregulation of IL-6 in the Ad-ME.TRAP+CoVaccineHT^TM^ group immediately post vaccination. It has been shown that IL-6 is involved in the protection against intracellular bacteria^23^ and is a pleotropic cytokine that regulates dendritic cell development, chemokine production, B cell development and antibody secretion, and T cell maturation. Importantly, it is a key mediator for the transition from innate to acquired immunity^24^.

Surprisingly, in the spleen, and to a degree in the peripheral blood, we observed a statistically significant reduction in the number of CD4^+^ and CD8^+^ T cells, but not γδ and NK cells, following the administration of the adjuvants. A similar reduction in Pb9-specific CD8^+^ T cells was detected in the peripheral blood, as well as liver and spleen, suggesting that the reduction in the circulating T cells was not simply a result of migration into other organs.

Protection from lethal disease in this animal model of malaria infection is conferred solely by an immunodominant CD8^+^ T cell epitope Pb9. However, in this study the frequency of Pb9-specific CD8^+^ T cells alone did not correlate with enhanced protection. Conversely, Ag-specific CD8^+^ T cell frequencies were markedly reduced in the peripheral blood, spleen and liver of the protected mice prior to the challenge. The inclusion of the CoVaccineHT^TM^ adjuvant resulted in a major shift towards Pb9-specific CD8^+^ T_CM_ responses in spleen and blood, and an increase in T_E_ responses in liver. This underlines the plasticity of CD8^+^ T cell responses in the presence or absence of an adjuvant and indicates the ability of CoVaccineHT^TM^ to generate a pool of central memory CD8^+^ T cells, which in mice have the capacity to convert to T_E_ and T_EM_ upon further antigenic stimulation^25^. Accelerated formation of memory T cell subsets has previously been linked to immunisation with peptide-loaded mature DCs^26^ and both of the protective adjuvants have been shown to enhance DC maturation^27, 28^.

Our analysis of CD8^+^ T cell functionality suggests that immunisation with adenoviral vector in combination with Abisco^®^-100 or CoVaccineHT^TM^ influences the quality of the T cell response by directing it towards a cytotoxic phenotype, characterized by the expression of the CD107a degranulation marker in the absence of IFNγ, IL-2 and TNFα. Degranulation is a major pathway in the CTL killing of viral or parasite infected cells and CD107a expression upon﻿ in vitro restimulation is now commonly used as a measure of antigen-specific cytolytic activity^29^. Expression of CD107a has previously been assessed following vaccination with adenoviral and MVA vaccine vectors^30^. Of interest, Capone *et al*. observed a similar induction of single positive CTL expressing CD107a only after 4 heterologous viral-vectored immunizations consisting on an Ad-ME.TRAP prime followed by three homologous boosts with Modified Vaccinia Ankara (MVA). As macaques cannot be challenged with *P*. *falciparum* malaria it was not possible to establish a correlation with efficacy in that study. Our results support our earlier observation in human volunteers immunised with the same vaccine prime-boost regimen and challenged with malaria, where single-positive CD107a CD8^+^ T cells were significantly associated with efficacy and long term protection^12^. Furthermore, a study by Bijker *et al*.^31^, in which healthy volunteers were immunised against malaria by exposure to *P*. *falciparum*-infected mosquito bites following chloroquine chemoprofilaxis, demonstrated that fully protected volunteers had a significantly higher proportion of CD4^+^ cells expressing CD107a and CD8^+^ cells producing Granzyme B.

In conclusion, we investigated the adjuvanting effect of 11 compounds on an adenovirus vectored vaccine in protection against malaria in a mouse model setting. Of the adjuvants tested, only two enhanced the vaccine-induced protection from pre-erythrocytic malaria, Abisco^®^-100  and CoVaccineHT^TM^. Analysis of antigen specific CD8^+^ T cell responses revealed that both adjuvants support the development of CD8^+^ T cells expressing the cytotoxic marker CD107a in the absence of IFNγ, TNFα and IL2, and CoVaccineHT^TM^ enhanced CD8^+^ T_CM_ responses in secondary lymphoid organs and peripheral blood. We demonstrate the ability of some adjuvants to improve the existing protective efficacy of viral vectored vaccines while enhancing CD8^+^ T cell responses; such compounds could contribute to the development of vaccine formulations able to achieve full protection against a disease after a single dose immunisation.

## Materials and Methods

### Mice and immunisations

#### Ethics Statement

All procedures were performed under the UK Home Office personal project licence PPL 30/2414, and approved by the University of Oxford Animal Care and Ethical Review Committee, in accordance with the terms of the UK Animals (Scientific Procedures) Act Project Licence.

Female BALB/c (H-2^d^) mice 6–8 weeks of age (Harlan Laboratories, Oxfordshire, UK) were anesthetized with Isofluorane (Isoflo, Abbot Animal Health, UK) prior to the immunizations. The immunisations were administered either intramuscularly (i.m.) into the hind limb or intradermally (i.d.) into the ear pinnae.

#### Viral vectors

Adenovirus and MVA vectors expressing the transgene ME.TRAP have been previously described^5, 14, 30^. The Ad-ME.TRAP construct was produced at the Clinical Biomanufacturing Facility at the Univesity of Oxford using starting plasmid material supplied by Okairos S.R.L., Italy, now part of GlaxoSmithKline. The insert ME.TRAP is a hybrid transgene of 2,398 bp encoding the *P*. *falciparum* 789 amino acid TRAP protein and a multiple epitope (ME) string, composed of a number of human B- and T cell epitopes as well as the BALB/c H-2K^d^ epitope Pb9^13, 15, 32^, which enables the assessment of the vaccine immunogenicity and efficacy in mice. Adenoviral vector used in this study was the chimpanzee adenovirus ChAd63-ME.TRAP. It was injected in mice, either i.d. or i.m., at the dose of 5 × 10^9^ vp (corresponding to around 5 × 10^7^ i.u.) which has previously been found to confer incomplete protection against malaria challenge.

#### Adjuvants

Adjuvants used in this study were obtained from a variety of sources and administered to mice at different doses. In all experiments, the vaccine was freshly formulated with the adjuvant and immunisations carried out within 2 hours. Different adjuvants were formulated with the viral vectored vaccine as follows: Abisco^®^-100, an ISCOM Matrix adjuvant (Isconova, Sweden, now Novavax, MD, USA), also known as Matrix-M, was mixed with the vaccine and briefly vortexed. Dose of Abisco^®^-100 per mouse was either 12 μg or 24 μg, as indicated in the Results section and the Figure legends. The mode of action of ISCOM adjuvants is currently subject of research; a mechanism independent of TLR4 has been suggested^33^. Alhydrogel^®^ (Brenntag, Denmark) at 85 µg of Al^3+^/dose was combined with Ag in TRIS buffered saline and rotated at RT for 30 min. before administration. Adjuvanting effect of alhydrogel has long been believed to relay on a depot-forming ability which would provide extended availability of the Ag. However in studies of excision of the immunisation site experimental data were not consistent with a depot effect^34^. Molecular mechanism of action of alhydrogel has been associated with the activation of the NAPL3 inflammasome^35^, although more recent papers have demonstrated an alternative, or additional, role in cross-linking and sorting of lipids in the cell membrane of antigen presenting cells^36^. CoVaccine HT^TM^ (British Techonology Group, BTG, UK), an oil-in-water vaccine adjuvant consisting of sucrose fatty acid sulphate esters (SFASE) immobilised on droplets of a squalane oil in water emulsion^37^, was combined with the vaccine in PBS by gentle pipetting. The dose of CoVaccineHT^TM^ per mouse was 1 mg. Mechanism of action of CoVaccineHT is currently unknown and remains subject of research. So far, activation of several human TLR receptors has been dismissed (Luuk Hilgers, personal communication). Two adjuvants based on a stable emulsion (SE) of squalene-like oil in water and containing one or more TLR agonists were obtained from the Infectious Disease Research Institute (IDRI), USA. Designated EM005 and EM014 and composed of SE containing TLR4 agonist glucopyranosyl lipid A (GLA) and SE containing GLA and TLR9 agonist CpG ODN 1826, respectively, the adjuvants were mixed with the vectored vaccines in PBS and vortexed for 30 seconds. The dose administered per mouse contained 20 µg of each TLR agonist. Adjuvants LS121, AQ016 and AL006 were also obtained from the IDRI, and administered following a simple admixture with the vectored vaccine and vortexing for 30 seconds. The dose per mouse of active ingredients in each adjuvant was as follows: LS121, 5 μg of GLA and 400 μg of liposomes consisting of DPPC (1,2-dipalmitoyl-sn-glycero-3-phosphocholine), DPPG (1,2-dipalmitoyl-sn-glycero-3-phosphoglycerol) and cholesterol; AL006: 100 μg of Aluminium hydroxide and 5 μg of GLA; AQ016: 5 μg of GLA. TiterMax^®^ Gold was obtained from Uptima-Interchim, France. It is a squalene oil-based water-in-oil adjuvant with added proprietary non-ionic surfactant block copolymer, CRL-8300. Its mode of action is not known. The cholera toxin (CT) adjuvant used was the B subunit of CT, pre-clinical grade (List Biological Laboratories, USA), supplied as lyophilised powder and reconstituted according to the manufacturer’s instructions. The adjuvant was mixed with the viral vectored vaccine by gentle pipetting and administered at doses and routes indicated in the Results and the Figures.

### Malaria parasite challenge

*Plasmodium berghei* (ANKA strain clone 234) sporozoites were isolated from salivary glands of female *Anopheles stephensi* mosquitoes. Parasites were resuspended in RPMI 1640 media and each mouse received a total of 1000 sporozoites via the i.v. route. Blood samples were taken daily between days 5–20 post challenge to measure parasitaemia: blood smears were stained with Giemsa and observed under a light microscope for the presence of parasites within the red blood cells. Survival was defined as a complete absence of parasites in blood, which is considered sterile protection.

### LIPS assay for determining antibody titres

The antibody titres against TRAP were assessed using the Luciferase Immunoprecipitation System (LIPS) assay^17^ as purified TRAP protein was not available. Briefly, HEK293 cells were transfected with a plasmid expressing the TRAP Ag fused to *Renilla* luciferase rluc8 gene and incubated overnight. Following harvesting, the cells were lysed, and luciferase expression levels in the cell lysate measured using a luminometer (Varioskan^®^ Flash, Thermo Scientific, UK). Serially diluted serum from immunised mice was incubated with the cell lysate containing 2 × 10^8^ LU/ml and then combined with Protein A/G UltraLink Resin (ThermoScientific, UK) on a filter HTS plate (Millipore, UK). The plate was washed to remove unbound antibodies and developed using the Renilla Luciferase Assay system (Promega, UK): *Renilla* luciferase substrate was added to each well, luminescence measured on the Varioskan and each well immediately quenched with 2 M HCl to prevent cross-talk between wells. Relative anti-TRAP antibody titres in each sample were expressed as light units of luminescence.

### Flow cytometry

Intracellular cytokine staining (ICS) was carried out using a standard protocol. Briefly, erythrocytes in whole blood pellets or fresh splenocytes were lysed with Ammonium-Chloride-Potassium buffer and mononuclear cells subsequently restimulated with the Pb9 peptide (1 μg/ml) by incubation at 37 °C for 5 hours in the presence of 1 μl/ml Golgi-Plug (BD Biosciences, UK). To assess the hepatic T cell responses, livers were firstly perfused with PBS to remove the circulating blood. Mononuclear cells were then isolated by mechanical disruption and incubation for 1 hour at 37 °C in FCS-free MEM media supplemented with glutamine (4 mM) and penicillin/streptomycin (100U penicillin/100μg streptomycin), containing DNase at a final concentration of 0.03 mg/ml and collagenase at 0.7 mg/ml (both from Sigma, UK). The digestion was stopped using MEM with 10% FCS, cells washed and lymphocytes purified with Ficoll-Paque Premium (GE Healthcare, UK) followed by *in vitro* stimulation with Pb9 peptide as described above.

Phenotypic analysis of CD8^+^ T cells was performed by surface marker and intracellular cytokine staining (ICS), using commonly employed antibodies (eBioscience, UK). Non-specific antibody binding was prevented by incubation with anti-CD16/CD32 Fcγ III/II Receptor (BD Pharmingen, UK) prior to the surface staining. Cells were permeabilised for intracellular staining using the BD Cytoperm/Cytofix^TM^ kit (BD Biosciences, UK). The Pb9 tetramer was produced by the NIH tetramer facility (MHC tetramer core facility, Emory University Vaccine Center, Atlanta, USA) using the peptide SYIPSAEKI (Proimmune, UK). Live and dead cells were distinguished using LIVE/DEAD^TM^ Fixable Aqua Dead Stain Kit (ThermoFisher Scientific, UK). Flow cytometric analyses were performed on LSRII (BD Biosciences, UK). Data were analyzed with either FACSDiva (BD) or FlowJo software. Analysis of multifunctional CD8^+^ T-cell responses was performed using a Boolean analysis in FlowJo and Pestle and SPICE 4.0 software, developed by M. Roederer (NIH, Bethesda)^18^.

### Cytokines in serum

Concentration of various cytokines in the serum of the immunised mice was assessed using the Th1/Th2/Th17/Th22 13-plex mouse multiplex bead array kit BMS822FF with added IL-1β simplex kit (both from eBioscience, UK), following the manufacturer’s protocol. The bead fluorescence was detected and measured using LSRII flow cytometer (BD Biosciences, UK) and data analysed with FlowJo software.

### Statistical Analysis

All statistical analyses were performed using appropriate statistical packages within the GraphPad Prism software, version 6. In malarial survival experiments, a Log Rank test was used to compare the level of significance between data sets. In other analyses, for non-parametric data Kruskal-Wallis test with Dunn’s multiple comparison post-test was used to compare more than two groups. One-way ANOVA was used for multiple comparisons of parametric data with Bonferroni’s multiple comparison post-test for comparison of groups as stated. An un-paired t-test was used to compare the means of two groups for parametric data and a Mann-Whitney U test was used for non-parametric data; p < 0.05 was considered significant (*p < 0.05, **p < 0.01 and ***p < 0.001).

## Electronic supplementary material

Supplementary Information
