# Publisher Correction: Adjuvanting a viral vectored vaccine against pre-erythrocytic malaria

**DOI:** 10.1038/s41598-019-43120-x

**Published:** 2019-05-03

**Authors:** Anita Milicic, Christine S. Rollier, Choon Kit Tang, Rhea Longley, Adrian V. S. Hill, Arturo Reyes-Sandoval

**Affiliations:** 1 The Jenner Institute, Nuffield Department of Medicine, University of Oxford, Old Road Campus Research Building, Roosevelt Drive, Oxford, OX3 7DQ UK; 2 Oxford Vaccine Group, Department of Paediatrics, University of Oxford and the NIHR Oxford Biomedical Research Centre, Centre for Clinical Vaccinology and Tropical Medicine, Churchill Hospital, Oxford, OX3 7LE UK; 3 The Jenner Institute, Nuffield Department of Medicine, University of Oxford, The Henry Wellcome Building for Molecular Physiology, Roosevelt Drive, Oxford, OX3 7BN UK; 4 Present Address: Duke-NUS Medical School, 8 College Road, Singapore, Singapore; 5 Present Address: Population Health and Immunity, Walter & Eliza Hall Institute, 1G Royal Parade, Parkville, VIC 3052 Australia and Mahidol Vivax Research Unit (MVRU) Faculty of Tropical Medicine, Mahidol University, Bangkok, 10400 Thailand

Correction to: *Scientific Reports* 10.1038/s41598-017-07246-0, published online 04 August 2017

In the original version of this Article, the author Christine S. Rollier was incorrectly indexed. This error has now been corrected.

