# Supplementary material for: Adjuvanting a viral vectored vaccine against pre-erythrocytic malaria
Source: Sci Rep. 2017 Aug 4;7:7284. doi: 10.1038/s41598-017-07246-0 (PMC5544665; doi:10.1038/s41598-017-07246-0)
Supplement: Supplementary file 1 — Supplementary Information [file 41598_2017_7246_MOESM1_ESM.pdf]

## Supplementary Figures S1 and S2

Adjuvanting a viral vectored vaccine against pre-erythrocytic malaria

*Milicic A, Rollier C, Tang CK, Longley R, Hill AVS and Reyes-Sandoval A.*

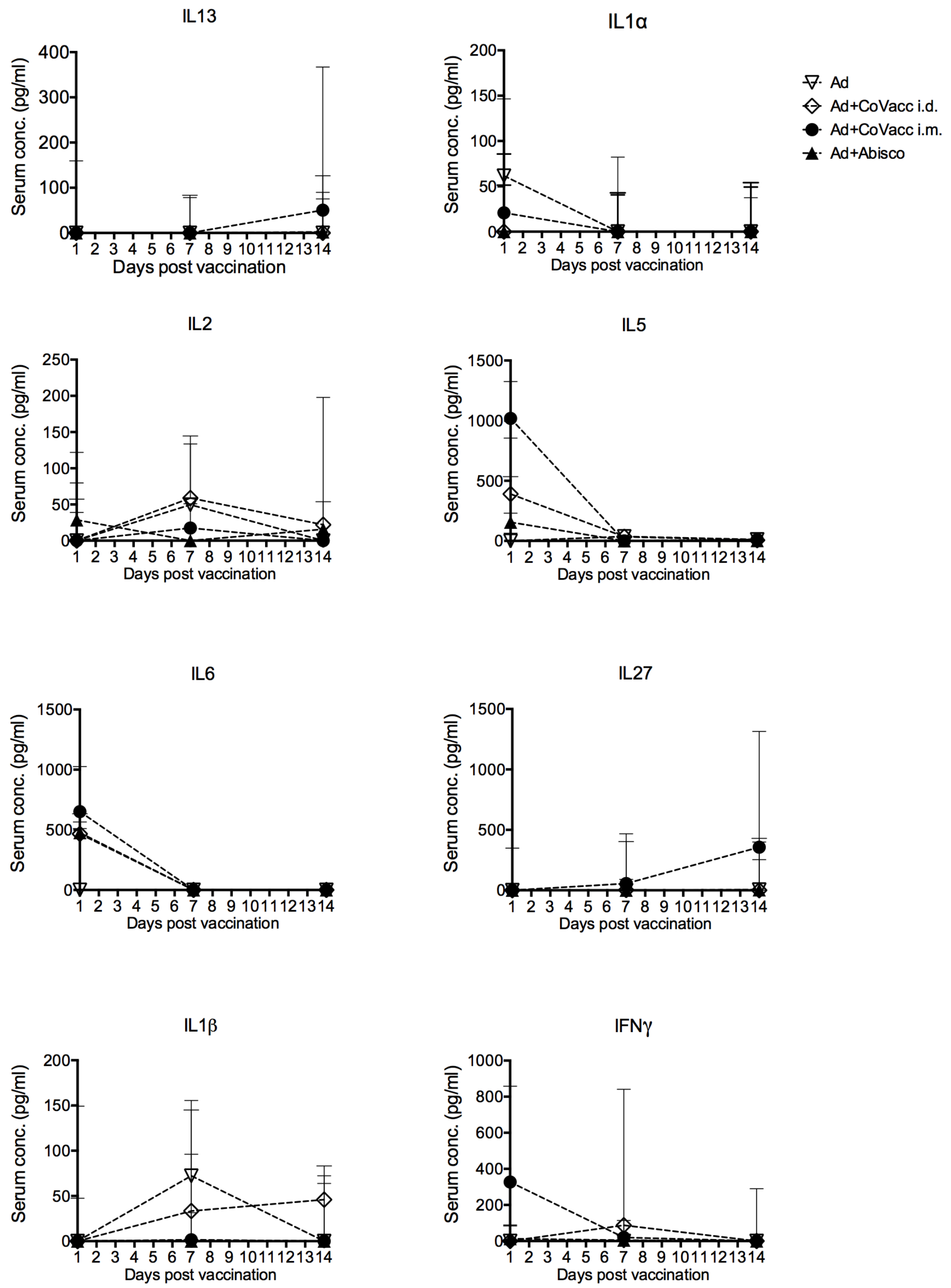


**Supplementary Figure S1: Analysis of serum cytokine levels in vaccinated animals.**
Serum samples from BALB/c mice (n=6), vaccinated with Ad-ME.TRAP vaccine ($5x10^9$ vp/dose) alone or adjuvanted with Abisco®-100 or CoVaccineHT, were collected at 1, 7 and 14 days post-immunisation. Serum cytokine levels were measured using Th1/Th2/Th17/Th22 13-plex mouse multiplex bead array kit with added IL-1β. Data are shown for all detected cytokines at all three time points.

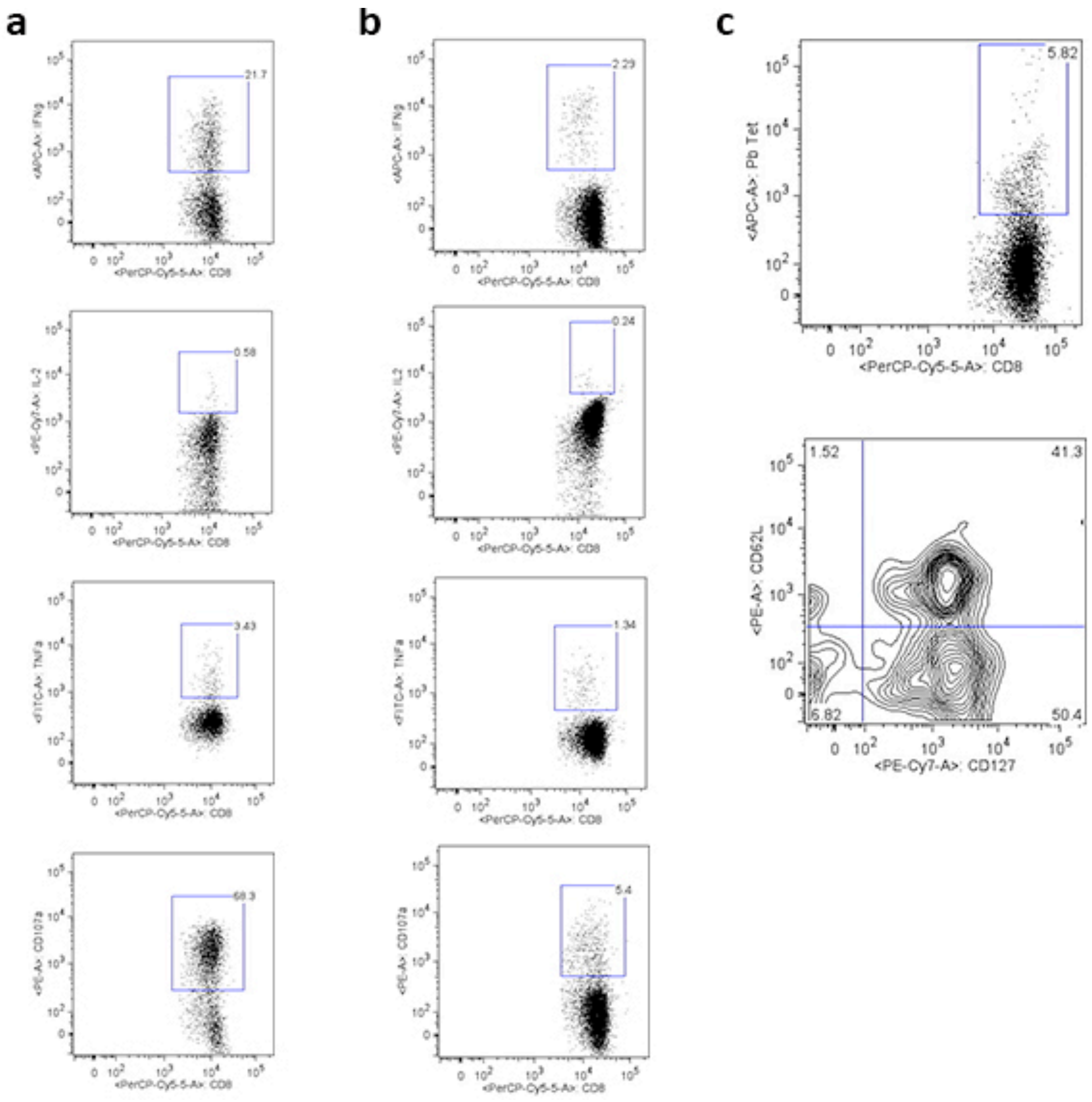


**Supplementary Figure S2: Representative FACS plots of the experiments using ICS.**

PBMCs (panels a and c) and splenocytes (panel b) from BALB/c mice (n=6), vaccinated with Ad-ME.TRAP vaccine ($5x10^9$ vp/dose) alone or adjuvanted with Abisco®-100 or CoVaccineHT, were collected at two weeks post- immunisation.

Production of IFNγ, TNFα and IL2 cytokines and degranulation (CD107a expression) by CD8+ PBMCs (panel a) or splenocytes (panel b), following 4h of in vitro stimulation with the Pb9 peptide, was assessed by intracellular staining and flow cytometry.

c) Proportion of Pb9-specific $CD8^+$ T cells in the peripheral blood, the spleen and the liver, and their memory profiles (effector, $T_E$, effector memory, $T_{EM}$ and central memory, $T_{CM}$) were evaluated by flow cytometry using the Pb9-tetramer and surface markers CD127 and CD62L. The three memory cell subsets were defined as: $T_E = CD62L^-CD127^-$, $T_{EM} = CD62L^-CD127^+$ and $T_{CM} = CD62L^+CD127^+$.
